# Vaccines for the prevention of diarrhea due to cholera, shigella, ETEC and rotavirus

**DOI:** 10.1186/1471-2458-13-S3-S11

**Published:** 2013-09-17

**Authors:** Jai K Das, Anjali Tripathi, Anum Ali, Amman Hassan, Chesarahima Dojosoeandy, Zulfiqar A Bhutta

**Affiliations:** 1 Division of Women & Child Health, The Aga Khan University, Karachi, Pakistan; 2 Global Child Health and Policy, Centre for Global Child Health, The Hospital for Sick Children, Toronto, ON, Canada

## Abstract

**Background:**

Diarrhea is a leading cause of mortality in children under 5 years along with its long-term impact on growth and cognitive development. Despite advances in the understanding of diarrheal disorders and management strategies, globally nearly 750,000 children die annually as a consequence of diarrhea.

**Methods:**

We conducted a systematic review of the efficacy and effectiveness studies. We used a standardized abstraction and grading format and performed meta-analyses for all outcomes. The estimated effect of cholera, shigella, Enterotoxigenic Escherichia coli (ETEC) and rotavirus vaccines was determined by applying the standard Child Health Epidemiology Reference Group (CHERG) rules.

**Results:**

A total of 24 papers were selected and analyzed for all the four vaccines. Based on the evidence, we propose a 74% mortality reduction in rotavirus specific mortality, 52% reduction in cholera incidence due to their respective vaccines. We did not find sufficient evidence and a suitable outcome to project mortality reductions for cholera, ETEC and shigella in children under 5 years.

**Conclusion:**

Vaccines for rotavirus and cholera have the potential to reduce diarrhea morbidity and mortality burden. But there is no substantial evidence of efficacy for ETEC and shigella vaccines, although several promising vaccine concepts are moving from the development and testing pipeline towards efficacy and Phase 3 trials.

## Introduction

Diarrhea is the leading cause of mortality in children under 5 years along with its long-term impact on growth and cognitive development. Despite advances in the understanding of diarrheal disorders and management strategies; globally 0.75 million children die annually as a consequence of diarrhea [1]. The number of cholera cases reported to World Health Organization (WHO) annually has remained relatively constant since 1995, varying from 100,000 to 300,000 cases per year [2]. An estimated 1 million deaths (60% in children under 5) and 165 million cases of dysentery annually were estimated to occur due to shigella [3], while more recent estimates place the shigella disease burden at about 90 million episodes and 108,000 deaths per year [2]. Enterotoxigenic Escherichia coli (ETEC) was thought to account for an estimated 200 million diarrhea episodes and 380,000 deaths annually [4], while a recent estimate suggests a lower figure of 170,000 deaths every year [2]. Rotavirus is the most common cause of severe dehydrating diarrhea in young children globally accounting for an estimated 527,000 (475 000-580 000) deaths each year, mostly in children under the age of two years [2]. Collectively these four organisms account for a great number of cases of diarrhea across the world and vaccines targeting the most common strains of all these pathogens are currently being developed, improved and undergoing trials across the globe.

Older generation injectable cholera vaccines have been abandoned since the 1970s owing to their limited efficacy and local side effects. Oral Cholera Vaccines (OCV) are good candidates for the control of cholera particularly in endemic areas. For example the inactivated or killed whole cell plus recombinant cholera toxin B subunit vaccine (rBS-WC) and the killed oral cholera vaccine (WC) has shown success in trials in Mozambique [5] and Vietnam respectively [6]. The potential for use of cholera vaccines is immense in public health, especially due to its herd immunity effect and recent research indicates that with this herd protection, even moderate coverage levels of targeted populations with killed OCVs may result in virtually complete control of cholera [7,8].

There are four different species and 47 antigenically distinct serotypes of Shigella, divided on the basis of differences in O antigen of their lipopolysaccharide which are *S. dysenteriae* (13 serotypes), *S. flexneri* (15 serotypes), *S. boydii* (18 serotypes), and *S. sonnei* (1 serotype). A safe and effective shigella vaccine offers great potential for controlling shigellosis and the potential of oral and parenteral shigella vaccines for conferring a high degree of serotype-specific immunity has been confirmed in some field trials [9-11]. The presence of 15 serotypes of *S. flexneri* is a barrier for vaccine development [12], although there is evidence of serologic cross-reactivity in humans [11] and cross-protection among *S. flexneri* serotypes in animals [13], suggesting that broad *S. flexneri* protection may be possible. In addition, recent pre-clinical animal studies also suggests that conserved Shigella invasion proteins may be able to induce broad protection against both *S. flexneri* and *S. sonnei* serotypes when given parenterally with a mucosal adjuvant [14].

Like Shigella, the incidence of ETEC infection peaks in early life and declines thereafter, suggesting that there may be natural immunity after repeated exposures [15,16]. ETEC associated morbidity is extremely high in many endemic countries with children experiencing 1-2 symptomatic episodes per child per year through the first 2-3 years of life. The high ETEC burden has also been associated with nutritional deficiency; both low weight for age as well as low height for age [16,17]. An inactivated whole cell ETEC vaccine designed to induce immune responses against both the Heat Labile toxin (LT)and common colonization factors protected travelers against more moderate to severe diarrhea in two trials (protective efficacy of ~70%), but did not confer protection among young children and infants in Egypt (protective efficacy of ~20%) [18,19].

Prevalence of rotavirus related gastro-enteritis is similar in developed and developing countries, showing that it has little or no association with sanitation systems, as opposed to the former three organisms. This makes vaccination even more important and studies have shown that vaccination contributes significantly towards reduction in cases of rotavirus related illnesses in the developed world. Efforts to develop rotavirus vaccine began in 1980s and were introduced in 1990s but were withdrawn after association with increased risk of intussusception, and this led to the development and testing of new vaccines for potential use in a range of settings. Recent Phase III trials of the monovalent rotavirus vaccine in Malawi and South Africa [20] have also shown a vaccine efficacy of 77% against severe rotavirus infection in South Africa and lower (50%) efficacy in Malawi. A previous review of six studies [21] based on the Lives Saved Tools (LiST) rules suggests that these vaccines are associated with a 74% reduction in rotavirus related diarrhea mortality but also indicated that protective efficacy may be lower in regions with high child mortality rates and residual burdens of bacterial diarrhea .

We conducted a systematic review followed by a meta-analysis of the published efficacy and effectiveness trials of vaccines against cholera, ETEC, shigella and rotavirus to provide a best possible impact of these interventions on cause specific mortality. We reviewed the available literature and evaluated the quality of included studies according to the Child Health Epidemiology Reference Group (CHERG) adaptation, Assessments, Development and Education (GRADE) criteria [22]. We performed new reviews for cholera, shigella and ETEC vaccines, while updated the existing LiST review on rotavirus vaccines [21].

## Methods

We systematically reviewed all published literature up to March 2012 to identify the efficacy and effectiveness studies describing the effects of vaccines related to cholera, shigella, ETEC and rotavirus in children less than or equal to 5 years. We specifically evaluated the evidence for vaccine related outcomes studies using recent recommendations from a GRADE working group on vaccines [23]. Following CHERG Systematic Review Guidelines, we searched PubMed, Cochrane Libraries, Embase and WHO Regional Databases and additional studies were identified by hand searching references of included studies. We used various combinations of *diarrhea*, *children*, *cholera*, *shigella*, *rotavirus*, *ETEC* and *vaccines* and included studies in any language.

### Inclusion criteria

Studies were included if they reported the effect of vaccines on morbidity and mortality associated with diarrhea due to cholera, shigella, ETEC and rotavirus in children under five years of age, as observed by morbidity and mortality outcomes. We also included studies that were conducted on a broader age group but had segregated data for the defined age group. We also included studies reporting on immunogenicity and adverse events outcomes. Only studies with a placebo group or a control group were included. Our original inclusion criteria included studies with children aged up to 5 years, however for shigella vaccines, we found one study in the defined age group hence we expanded our inclusion criteria to include children up to sixteen years of age.

### Abstraction, analysis and summary measure

We abstracted data for studies that met the final inclusion and exclusion criteria. Data describing study identifiers and context, study design and limitations, intervention specifics and outcome effects, were abstracted into a standardized abstraction sheet as detailed in the CHERG Systematic Review Guidelines. Each study was assessed and graded according to the CHERG adaptation of the GRADE technique. Randomized trials received an initial score of “high”. We deducted a grade point for each study design limitation.

### Quantitative data synthesis

We conducted a meta-analysis for individual studies and pooled statistics was reported as the relative risk (RR) between the experimental and control groups with 95% confidence intervals (CI). Mantel–Haenszel pooled RR and corresponding 95% CI were reported or the DerSimonian–Laird pooled RR and corresponding 95% CI where there was an unexplained heterogeneity. All analyses were conducted using the software Review Manager 5.1. Heterogeneity was quantified by Chi^2^ and I^2^, which can be interpreted as the percentage of the total variation between studies that is attributable to heterogeneity rather than to chance, a low p-value (less than 0.1) or a large chi-squared statistic relative to its degree of freedom and I^2^ values greater than 50% were taken as substantial and high heterogeneity. In situations of high heterogeneity, causes were explored by sensitivity analysis and random effect models were used.

We summarized the evidence by outcome, including assessments of study quality and the quantitative measures, according to the standard guidelines. A grade of “high”, “moderate”, “low” and “very low” was used for grading the overall evidence indicating the strength of an effect on specific health outcome according to the CHERG Rules for Evidence Review.

## Results

### Cholera

**Figure 1:**
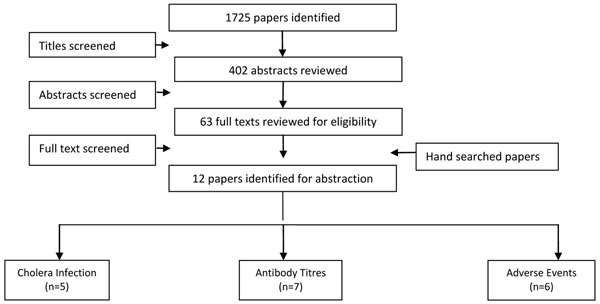
Search strategy flow chart for Cholera

We identified 1725 titles from search conducted in all databases. After screening titles and abstracts, 12 [5,6,23-33] studies were identified that met the inclusion criteria (Figure 1). Ten of the studies were Randomized Controlled Trials (RCTs) while one was a quasi-experimental design and one was a case control study. All of these studies were conducted in developing countries and had evaluated the effect of oral vaccines including WC, BS-WC and live oral vaccines. Greater than two folds rise in the vibriocidal antibody titres was reported by seven studies and it showed a significant increase (RR of 2.24, 95% CI: 1.32, 3.80). Sub group analysis showed that killed oral vaccines were associated with a non-significant rise in vibriocidal antibody titres (RR: 0.97, 95% CI: 0.75, 1.25), while live oral vaccines were associated with a significant rise (RR: 10.73, 95% CI: 1.94, 59.33). For assessment of outcomes related to the incidence of cholera, we reviewed both effectiveness and efficacy trials. Three data sets from two studies showed that cholera vaccination with a one year follow up were associated with a 52% reduction (RR: 0.48, 95% CI: 0.35, 0.64) in the incidence of cholera based on the effectiveness trials, while analysis of efficacy studies showed a non-significant reduction of 10% (RR: 0.90, 95% CI: 0.40, 2.03). While sub group analysis based on vaccine type, showed that; WC was associated with a 47% reduction (RR: 0.53, 95% CI: 0.36, 0.76), BS-WC was associated with a 53% reduction (RR: 0.47, 95% CI: 0.30, 0.74) and live oral CVD 103-Hg R with a non-significant impact (RR: 1.00, 95% CI: 0.20, 5.00) on the incidence of cholera at one year follow up, although only a single study was pooled for the latter two. Adverse events just after the vaccination were also pooled and included fever, nausea, vomiting, abdominal pain and diarrhea with a RR of 1.42 (95% CI: 1.06, 1.89) (Table 1). Given the design and standard of care in RCTs it was not possible to assess mortality impact.

**Table 1:** Quality assessment of vaccine trials for immunization against – cholera

	Quality Assessment					Summary of Findings		
				Directness		No of events		

No of Studies	Design	Limitations	Consistency	Generalizability to population of interest	Generalizability to intervention of interest	Intervention	Control	Risk Ratio

*Effectiveness against morbidity*, *Cholera Infection:* Low*-outcome-specific quality*								

02 [6,32]	RCT/Quasi	One study was a Quasi Experimental Design	All studies show consistent benefit	Results can be generalised to population in developing countries	Included WC and BS-WC oral vaccines	63	75	0.48 [0.35, 0.64]^a^
O1 [5]	Case Control	Random model used		Study was conducted in Mozambique	BS-WC vaccine	2	22	0.18 (0.03, 1.08)

*Efficacy against morbidity*, *Cholera Infection: High-outcome-specific quality*								

02 [29,33]	RCT		All studies show consistent benefit	Results can be generalised to population in developing countries	Included WC and CVD 103-HgR live oral Vaccines	11	13	0.90 [0.40, 2.03]^a^

Efficacy/Effectiveness against morbidity- Cholera Infection (Various Types of vaccines)								

03 [6,32,33]	RCT/Quasi	One study was a quasi-experimental design	Two studies showed significant impact	Results can be generalised to population in developing countries	WC Vaccines	45	85	0.53 [0.36, 0.76]^b^
01 [32]	RCT	Only one study		Study was conducted in Bangladesh	BS-WC Vaccines	26	56	0.47 [0.30, 0.74]
01 [29]	RCT	Only one study		Study was conducted in Indonesia	CVD 103-HgR live oral	03	03	1.00 [0.20, 5.00]
*Vibriocidal antibody: Low outcome-specific quality*								

07 [23,25,27-30,65]	RCT	None	Results from analysis significant. Five studies show benefit	All from Developing Countries	All Oral Vaccines	503	234	2.24 (1.32, 3.80)^b^
03 [23,27,31]	RCT		Two studies show benefit	All from Developing Countries	Oral Killed Vaccines	202	205	0.97 [0.75, 1.25]^b^
04 [25,28-30]	RCT		Three studies show significant benefit	All from Developing Countries	Oral Live Vaccines	301	29	10.73 [1.94, 59.37]^b^

*One or more Adverse effect: Low outcome-specific quality*								

06 [24,25,27,28,30,32,33]	RCT	None	significant results	All from developing countries	All Oral Vaccines	132	91	1.42 (1.06, 1.89)^a^

a: Fixed Effect Model

b: Random Effect Model

### Shigella

**Figure 2:**
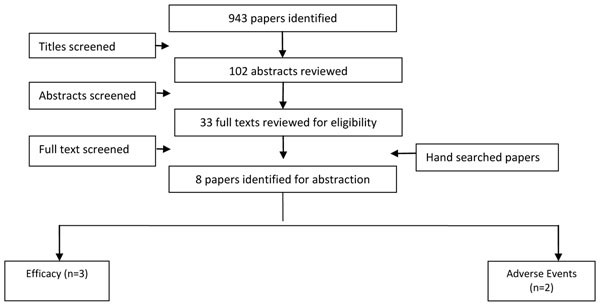
Search strategy flow chart for Shigella

It is recognized that there are no commercially licensed shigella vaccines, although several candidates have been studied in humans. A total of eight [34-41] studies were selected which met the inclusion criteria (Figure 2). All were RCTs and three were from developing countries. Vaccines for two shigella strains (*shigella flexneri* and *shigella sonnei*) were evaluated by these studies. No study reported on the outcome of mortality, while three reported on morbidity; which was the incidence of shigella. The analysis showed that shigella vaccine for *S.flexneri* was associated with a non-significant 28% reduction (RR: 0.72, 95% CI: 0.37, 1.39), while vaccines for *S.sonnei* were associated with a non-significant 53% reduction (RR: 0.47, 95% CI: 0.12, 1.85) in the incidence of *S.flexneri* and *S.sonnnei* respectively. Subgroup analysis based on the type of vaccines (oral and parenteral) also showed non-significant results for both the vaccines. Two studies reported on adverse events after vaccination and did not show any significant excess (RR 1.58, 95% CI: 0.81, 3.07) (Table 2).

**Table 2:** Quality assessment of vaccine trials for immunization against – Shigella

	Quality Assessment					Summary of Findings		
				Directness		No of events		

No of Studies	Design	Limitations	Consistency	Generalizability to population of interest	Generalizability to intervention of interest	Intervention	Control	Risk Ratio

*Efficacy against morbidity*, *Shigella Infection* (*S.Flexneri*)*:* Low*-outcome-specific quality*								

03 [35,38,40]	RCT	Random effect model. Insignificant results	Two studies show consistent benefit	Two studies were from developed countries	Two used oral vaccine while one used intramuscular	56	107	0.72 (0.37, 1.39)^b^
02 [35,38]	RCT	Random effect model. Insignificant results	One study shows significant benefit	One study from developed country	Oral Vaccines	49	99	0.67 [0.28, 1.59]^b^
01 [40]	RCT	Only one study		Study was conducted in Israel	Parenteral Vaccine	07	08	0.92 [0.33, 2.53]

*Efficacy against morbidity*, *Shigella Infection* (*S.Sonnei*)*: Low-outcome-specific quality*								

03 [35,38,40]	RCT	Random effect model. Insignificant results	Two studies show consistent benefit	Two studies were from developed countries	Two used oral vaccine while one used intramuscular	39	94	0.47 (0.12, 1.85)^b^
02 [35,38]	RCT	Random effect model. Insignificant results	One study shows significant benefit	One study from developed country	Oral Vaccines	10	56	0.39 [0.04, 4.33]^b^
01 [40]	RCT	Only one study		Study was conducted in Israel	Parenteral Vaccines	29	38	0.73 [0.45, 1.17]

*One or more Adverse events* (*S.Flexneri*)*: Low outcome-specific quality*								

02 [36,41]	RCT	None	inconsistent results	Both from developing countries		45	08	1.58(0.81, 3.07)^a^

a: Fixed Effect Model

b: Random Effect Model

### ETEC

**Figure 3:**
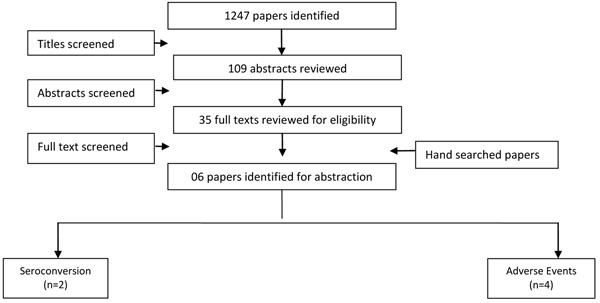
Search strategy flow chart for ETEC

Similar to Shigella, there are no licensed ETEC vaccines. We identified 1247 titles from our search of which only six [42-47] studies were reviewed that matched our inclusion criteria (Figure 3). Five of the identified studies were RCTs. All of these were conducted in either Egypt or Bangladesh. Studies provided data on immunogenicity and adverse events but expectably, none had information on morbidity or mortality . Two studies were pooled for IgA seroconversion with a RR of 2.70 (95% CI: 1.87, 3.90) and two for IgG seroconversion with a RR of 4.99 (95% CI: 2.51, 9.92). Adverse events after the vaccination had a RR of 1.58 (95% CI: 1.4, 2.19) (Table 3).

**Table 3:** Quality assessment of vaccine trials for immunization against – ETEC

Results for ETEC			
Outcomes	No of Studies	No of Participants	Impact Estimates (95% CI)

Serum Ig A seroconversion	2 [42,47]	157	2.70 [1.87, 3.90]^a^
Serum Ig G seroconversion	2 [42,47]	157	4.99 [2.51, 9.92]^a^
Adverse Events	4 [43,44,46,47]	1169	1.58 (1.14, 2.19)^a^

a: Fixed Effect Model

b: Random Effect Model

### Rotavirus

For rotavirus vaccine, we updated the previous review and ran a search after the last search date and found five new efficacy studies [48-52] which were included and no new effectiveness studies were found. We identified new studies reporting on outcomes of rotavirus hospitalizations (n=2); diarrhea hospitalizations (n=2); severe rotavirus gastroenteritis (n=5); severe diarrhea (n=4); and rotavirus gastroenteritis of any severity (n=3). All the new data was entered with the previous estimates and reanalyzed. There was no change in the effectiveness outcomes as no new study was identified while the new estimates for the efficacy outcomes are reported in table 4. Results from two new large studies from Bangladesh and India are expected within 2013 and should provide much needed information on the effectiveness of rotavirus vaccines in South Asia. In the interval surveillance data from several countries in Latin America, notably from Mexico [54] does show a significant impact of childhood diarrhea mortality since the introduction of the rotavirus vaccine.

**Table 4:** Quality assessment of vaccine trials for immunization against – Rotavirus

	Quality Assessment					Summary of Findings		
				Directness		No of events		

No of Studies	Design	Limitations	Consistency	Generalizability to population of interest	Generalizability to intervention of interest	Intervention	Control	Relative Risk (95% CI)

*Effectiveness against very severe rotavirus infection: Moderate/low outcome-specific quality*								

One	Matched case control	Hospital-based surveillance for cases	NA	Urban and peri-urban hospitals in Nicaragua	Pentavalent vaccine	43	255	74% (35–90%)

*Effectiveness against severe rotavirus infection: Moderate/low outcome-specific quality*								

One	Matched case control	Hospital-based surveillance for cases	NA	Urban and peri-urban hospitals in Nicaragua	Pentavalent vaccine	155	926	61% (38–75%)

*Effectiveness against rotavirus hospitalizations: Moderate outcome-specific quality*								

One	Matched case control	None	NA	Urban and peri-urban hospitals in Nicaragua (-0.5)	Pentavalent vaccine	216	1250	47% (22–64%)
One	Matched case control	None	NA	Rural hospital in the Northern Territory of Australia	Monovalent vaccine	10	58	57% (<0–83%)

*Efficacy against severe rotavirus infection: High outcome-specific quality*								

Eight	RCT	None	Heterogeneity from meta-analysis all studies show benefit	Four studies from developing and four from developed countries	Two used Monovalent Vaccine	237	745	0.17 [0.09, 0.32]^b^

*Efficacy against severe GI infection: Moderate outcome-specific quality*								

Six	RCT	None	All studies show benefit	Two from developed countries	Two used Monovalent Vaccine	1328	1573	0.68 [0.57, 0.81]^b^

*Efficacy against rotavirus hospitalizations: Moderate outcome-specific quality*								

Five	RCT	None	All studies show benefit	Four studies from developed countries	Two used Monovalent Vaccine	46	329	0.11 [0.05, 0.27]^b^

*Efficacy against GI hospitalizations: Moderate outcome-specific quality*								

Two	RCT	None	All studies show benefit	USA, Europe and Latin America	Two of three studies used monovalent vaccine; one used pentavalent.	203	607	0.43 (0.21, 0.9)^b^

*Efficacy against any rotavirus: High outcome-specific quality*								

Five	RCT	None	All studies show benefit	Four studies from developed countries	One study used monovalent vaccine	586	1348	0.39 [0.25, 0.61]^b^

a: Fixed Effect Model

b: Random Effect Model

### Recommendation for the LiST model

The principal objective of our exercise was to provide some estimates of vaccine effectiveness (for current and potential future vaccines) on childhood diarrhea and mortality for LiST. While we applied the CHERG rules for evidence review to available outcomes, there were several limitations in the available trial data. For cholera vaccines, there was no mortality data so we used estimates based on reduction in the frequency of severe cholera-associated morbidity, which is the cholera incidence to estimate a 52% reduction in the incidence of cholera after vaccination against cholera. For rotavirus vaccines, we did not find any further effectiveness trial hence the recommendation stays the same that is a 74% reduction in rotavirus specific mortality based on severe morbidity. For ETEC vaccines we did not find any outcome which could be projected to estimate the effect of ETEC vaccines on mortality although several trials of ETEC vaccines in travelers indicated that they have their greatest impact against moderate to severe disease [18,19]. This may be an important outcome to assess in future ETEC efficacy and effectiveness studies once promising vaccine currently under development move into this stage of field testing. For shigella vaccines, currently there is no evidence against mortality and the evidence on protective efficacy against illness is also limited, like ETEC, in the context of modeling for LiST.

## Discussion

Diarrheal diseases continue to claim approximately 0.75 million lives of children below the age of 5 [1]. The availability and usage of vaccines against four of the most common pathogens of diarrhea could help in significantly reducing this burden, although the development process for all vaccines is unequal. Our analysis shows that while one could potentially reduce a substantial number of rotavirus associated deaths and possibly for cholera in appropriate contexts, the same cannot be said for shigella and ETEC. The current data on the latter two vaccines is hampered by lack of licensed vaccines for public health use and adequate trials to assess efficacy in appropriate settings particularly against more moderate to severe form of illnesses.

We were able to generate potential estimates for efficacy/effectiveness for the two licensed vaccines based on current data. We estimated a summary impact estimate of 52% reduction in the incidence of cholera with the use of cholera vaccines. Although the quality of evidence was low and only two studies were evaluated, both were from endemic populations in developing countries and from effectiveness trials. The vaccines evaluated included oral WC and BS-WC which are currently available and licensed and can be potentially used among susceptible populations. For rotavirus vaccine we estimated that currently marketed rotavirus vaccines could prevent 74% (35–90%) of rotavirus deaths and 47–57% of rotavirus hospitalizations but variability in efficacy in various geographic settings is recognized. Rotavirus vaccines, thus, have the potential to greatly reduce the fraction of diarrhea deaths due to rotavirus. These estimates will be revised once the newer data from trials in south Asia become available. We were unable to estimate any effect sizes for mortality reduction with potential shigella and ETEC vaccines as much of the data from experimental vaccines is on immunogenicity with no evidence of impact on disease burden.

Notwithstanding the need to integrate preventive strategies against common diarrhea pathogens and disease burden, vaccination strategies are important adjunctive interventions and could impact mortality and morbidity [53,54]. While the use of rotavirus vaccines has been recommended by WHO [57], the potential role and usage of oral cholera vaccines as an adjunct to the control of cholera in endemic areas and during outbreaks is unclear. The reformulation of a bivalent WC oral vaccine is an affordable and safe for use in cholera endemic areas and can be an exciting development. Our meta-analysis of children under 5 years of age shows a significant 52% reduction in the number of cholera cases by the use of the vaccine in endemic populations.

The case for other vaccines is less clear because of limited information. Only a limited number of ETEC vaccine trials that have been conducted among younger age groups in endemic areas with only one Phase 3 efficacy trial which was also not able to determine the vaccines impact against more severe or life threatening ETEC disease [18,19]. ETEC vaccine has shown to be a feasible strategy based on the observation that immunity may be acquired through natural infections, [55,56] which is further supported by the observed decrease in ETEC-related events after the first year of life where ETEC infections are prevalent [57,58]. There are various challenges concerning the development of an effective and safe vaccine for children in developing countries, the most significant being the reduced immunogenicity and protective efficacy of oral vaccines in this population [59-62]. Despite such limitations, the analysis of the ETEC-rCTB vaccine suggests that it is immunogenic and relatively safe. Unpublished data from an efficacy trial among Egyptian children showed limited benefit, although the WHO recommended further testing after reformulation of the vaccine to contain more CFA antigens and modification of the surveillance approach to capture impact on more severe forms of ETEC infections [63]. A re-formulated version of this vaccine containing more CFA’s per dose has recently gone back into clinical trials with the new mucosal adjuvant, dmLT [18].

Like for ETEC, there are currently no licensed Shigella vaccines despite their development being a WHO priority for over 20 years. An expert panel convened by the Child Health and Nutrition Research Initiative (CHNRI) of the World Bank identified *Shigella* one of the highest priorities for long-term vaccine development [64]. In practice, the greatest impact on mortality of enteric vaccines, like Shigella, will be seen in medically underserved populations [65] and a safe, effective, and practical *Shigella* vaccine would likely save tens of thousands of lives and prevent hundreds of thousands of diarrheal illnesses in the developing world and in international travelers. Limited field trials of early live attenuated vaccine candidates or more recently, trials of polysaccharide based conjugate vaccines have shown vaccination to be a potentially effective public health tool in disease prevention and control [39,40] but currently there are no commercial products in the market. However, there are several promising candidates in the development pipeline, including the evaluation of conserved Shigella invasion proteins that may help broaden vaccine coverage [66].

The use of vaccines seems a more applicable near-term solution due to its potential cost-effectiveness, and thus constitutes a promising alternative strategy. While the results have so far been quite favorable, current research on vaccines is still quite limited, though it is said to represent more activity in the field than we have previously seen [67]. For example, there is currently only one vaccine that is at the forefront of pediatric ETEC research: the ETEC/rCTB vaccine, similarly more emphasis has been laid on the killed oral cholera vaccine than on any other form of the vaccine, with a majority of studies coming in from Bangladesh and Vietnam, hampering the generalizability of results, with similar limitations observed in studies with shigella and rotavirus as well. It is therefore important to underscore the importance of continued research in varying contexts to help refine and define global policies for the use of vaccines for the control of diarrheal disorders.

## Abbreviations

CHERG: Child Health Epidemiology Reference Group; CI: Confidence Intervals; ETEC: Enterotoxigenic Escherichia coli (ETEC); GRADE: Grading of Recommendations, Assessments, Development and Education; LiST: Lives Saved Tools; OCV: Oral Cholera Vaccines; rBS-WC: killed whole cell plus recombinant cholera toxin B subunit vaccine; RCTs: Randomized Controlled Trials; RR: Relative Risk; WC: killed oral cholera vaccine; WHO: World Health Organization.

## Conflict of interests

The authors declare that they have no conflict of interest.

## Authors' contributions

Dr ZAB was responsible for designing the review and co-ordinating the review with JKD. AT, AH, AL and CD were responsible for: data collection, screening the search results, screening retrieved papers against inclusion criteria, appraising quality of papers, abstracting data from papers, entering data into RevMan and analysis for shigella vaccines, cholera vaccines, rotavirus vaccines and ETEC vaccines respectively. ZAB and JKD supervised the project, interpreted the data and wrote the review. ZAB critically reviewed and modified the manuscript.

## Declaration

The publication costs for this supplement were funded by a grant from the Bill & Melinda Gates Foundation to the US Fund for UNICEF (grant 43386 to "Promote evidence-based decision making in designing maternal, neonatal, and child health interventions in low- and middle-income countries”). The Supplement Editor is the principal investigator and lead in the development of the Lives Saved Tool (LiST), supported by grant 43386. He declares that he has no competing interests.

This article has been published as part of *BMC Public Health* Volume 13 Supplement 3, 2013: The Lives Saved Tool in 2013: new capabilities and applications. The full contents of the supplement are available online at http://www.biomedcentral.com/bmcpublichealth/supplements/13/S3.
